# Exploring the immune landscape and drug prediction of an M2 tumor‐associated macrophage‐related gene signature in EGFR‐negative lung adenocarcinoma

**DOI:** 10.1111/1759-7714.15375

**Published:** 2024-06-17

**Authors:** Yajie Huang, Yaozhong Zhang, Xiaoyang Duan, Ran Hou, Qi Wang, Jian Shi

**Affiliations:** ^1^ Department of Medical Oncology The Fourth Hospital of Hebei Medical University Shijiazhuang China; ^2^ Department of Infectious Diseases The Fourth Hospital of Hebei Medical University Shijiazhuang China; ^3^ Department of Endoscopy The Fourth Hospital of Hebei Medical University Shijiazhuang China

**Keywords:** bulk RNA‐seq, EGFR‐negative lung adenocarcinoma, immune landscape, single‐cell RNA‐seq, tumor‐associated macrophage

## Abstract

**Background:**

Improving immunotherapy efficacy for EGFR‐negative lung adenocarcinoma (LUAD) patients remains a critical challenge, and the therapeutic effect of immunotherapy is largely determined by the tumor microenvironment (TME). Tumor‐associated macrophages (TAMs) are the top‐ranked immune infiltrating cells in the TME, and M2‐TAMs exert potent roles in tumor promotion and chemotherapy resistance. An M2‐TAM‐based prognostic signature was constructed by integrative analysis of single‐cell RNA‐seq (scRNA‐seq) and bulk RNA‐seq data to reveal the immune landscape and select drugs in EGFR‐negative LUAD.

**Methods:**

M2‐TAM‐based biomarkers were obtained from the intersection of bulk RNA‐seq data and scRNA‐seq data. After consensus clustering of EGFR‐negative LUAD into different clusters based on M2‐TAM‐based genes, we compared the prognosis, clinical features, estimate scores, immune infiltration, and checkpoint genes among the clusters. Next, we combined univariate Cox and LASSO regression analyses to establish an M2‐TAM‐based prognostic signature.

**Results:**

CCL20, HLA‐DMA, HLA‐DRB5, KLF4, and TMSB4X were verified as prognostic M2‐like TAM‐related genes by univariate Cox and LASSO regression analyses. IPS and TMB analyses revealed that the high‐risk group responded better to common immunotherapy.

**Conclusion:**

The study shows the potential of the M2‐like TAM‐related gene signature in EGFR‐negative LUAD, explores the immune landscape based on M2‐like TAM‐related genes, and predict immunotherapy response of patients with EGFR‐negative LUAD, providing a new insight for individualized treatment.

## Graphical abstract

The process of M2 Tumor‐Associated Macrophage‐Related Gene Signature Construction.

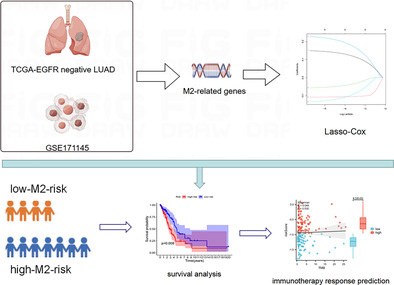

## INTRODUCTION

The China National Cancer Center published the latest data that one fifth of new cancer cases are in patients with lung cancer.^1^ In addition, lung adenocarcinoma (LUAD) is the main cause of death in cancer patients.^2^, ^3^ Although immune checkpoint inhibitors (ICIs) prolong the overall survival rate/time of advanced‐LUAD patients,^4^, ^5^ improvements are still needed in the era of LUAD prognosis. Asia has been reported to have the highest epidermal growth factor receptor (EGFR) prevalence (40%–55%),^6^, ^7^, ^8^, ^9^ followed by South America (36%), and the Caucasian population (19.7%).^10^, ^11^ The Oceania lung adenocarcinoma patients, which only included data from Australia, had the lowest *EGFR* mutation frequency at 12%. In another study, the *EGFR* mutation frequency of African lung adenocarcinomas patients was only around 8%.^10^ In particular, patients without *EGFR* mutations comprise nearly 50% of the total LUAD population. Previous studies have reported that patients with LUAD EGFR‐negative tumors show a higher response to ICIs,^12^ which may be largely determined by the complex tumor immune microenvironment (TME) in LUAD.^13^ Thus, we explored in great depth an innovative “signature” based on the TME in EGFR‐negative LUAD to provide new insight into prognosis prediction and possible drug selection.

TME is a potential therapeutic target which can forecast ICI response.^14^ Furthermore, studies on the TME of LUAD have suggested that immune cell infiltration is closely associated with progression and immunotherapeutic response.^15^, ^16^, ^17^, ^18^ Macrophages compose the main component of infiltrating immune cells in the TME, and tumor‐associated macrophages (TAMs) may be a predictive factor of cancer prognosis.^19^ Classically activated macrophages (M1) and alternatively activated macrophages (M2) are two main subtypes of macrophages. M2 macrophages can be activated by IL10 or Arg1 and then suppress antitumor immunity;^20^ furthermore, high M2/M1 ratio is an independent risk factor in prognosis of many cancers.^21^ Previous studies have built an M2‐like TAM‐related prognostic model for melanoma and explored the role of VARS1 in melanoma progression and M2 macrophage polarization. Therefore, it is meaningful to explore molecular characteristics combining M2‐like TAMs infiltration and to ascertain the significant factors of M2‐like TAM polarization.

The development of single‐cell RNA‐sequencing (scRNA‐seq) technology and associated methods for data analysis has provided an unprecedented opportunity to unravel the molecular characteristics of diverse immune cell populations in the TME. In order to bring new insights into the molecular characteristics of TAM infiltration in EGFR‐negative LUAD, the molecular markers of TAMS were identified based on the scRNA‐seq dataset. Then, we used weighted gene coexpression network analysis (WGCNA) to identify the M2‐like TAM hub molecular markers in the TCGA‐EGFR‐negative LUAD dataset. Next, we obtained the M2‐like TAM related markers by intersecting the two above mentioned markers. After that, we constructed a M2‐like TAM related signature according to the M2‐like TAM related markers and compared the prognosis, immune cell infiltration, and immunotherapy response in the high‐ and low‐risk groups. Therefore, we performed in‐depth research focusing on the effect of M2‐like TAMs on EGFR‐negative LUAD.

## METHODS

### Acquisition and single‐cell analysis

**FIGURE 1:**
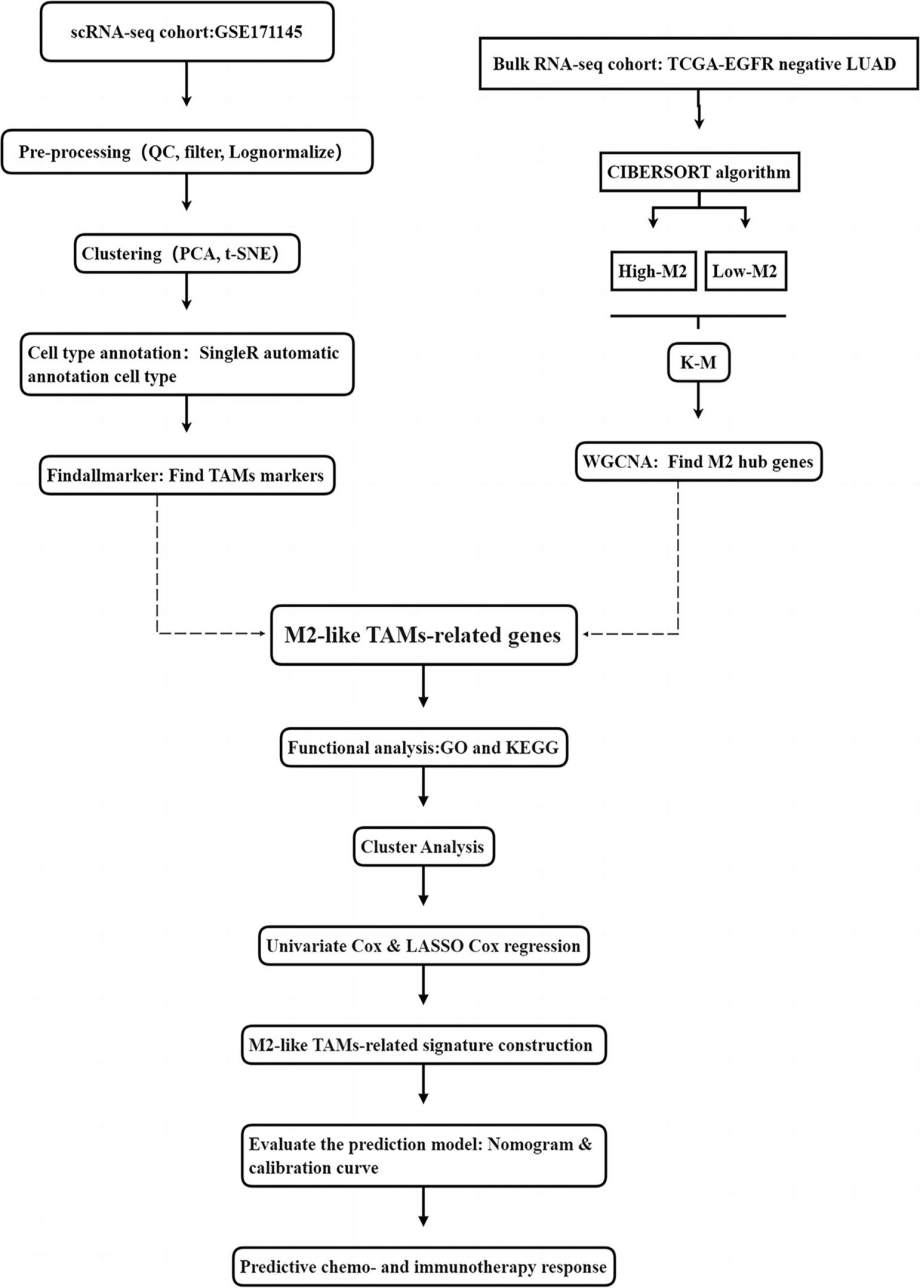
The work flow of the study.

Bulk transcriptome data and clinical features of LUAD were downloaded from the Cancer Genome Atlas (TCGA, https://portal.gdc.cancer.gov/), and we then screened 191 EGFR‐negative LUAD cases according to clinical data. The data were normalized to log_2_(value + 1). Single‐cell RNA‐seq (scRNA‐seq) data were extracted from the Gene Expression Omnibus (GEO, https://www.ncbi.nlm.nih.gov/geo/): GSE171145 (4 EGFR‐negative LUAD cases were included). Seurat^22^ was applied to analyze the scRNA‐seq data. First, a data quality check was done, and inferior quality cells with detected genes less than 200 or mitochondrion‐derived genes more than 5% were excluded. After normalizing the data using the logNormalize method, principal component analysis (PCA) was performed for essential principal component (PC) identification, and t‐distributed stochastic neighbor embedding (t‐SNE) was subsequently applied for cell cluster division. SingleR^23^ was used for cell type annotation and FindAllMarkers to identify TAMs‐genes by setting min.pct = 0.25. The workflow of the study is shown in Figure 1.

### Identification of M2‐like TAM‐related genes

The proportions of M2 macrophages in each EGFR‐negative LUAD sample from TCGA were obtained by the CIBERSORT algorithm.^24^ Next, the 191 EGFR‐negative LUAD samples were grouped into high‐ or low‐M2 macrophage infiltration teams according to the best cutoff value calculated by X‐tile.^25^ Yates' correction was used to compare the clinical significance between the high‐ or low‐M2 groups. Prognosis between the low‐ and high‐M2 macrophage infiltration groups was compared by Kaplan–Meier analysis. Then, the “WGCNA” way^26^ was applied to screen out M2 macrophage hub genes. First, hierarchical clustering was carried out on the cases to calculate outliers and remove abnormal cases. The soft threshold was selected by pickSoftThreshold. Afterwards, an adjacency matrix was constructed and converted into a topological overlap matrix (TOM), and the degree of dissimilarity constructed a gene dendrogram and module color. The minimum number of genes in each module was set to 60, and nine modules were produced. Correlations between modules and high‐ or low‐infiltrating M2 macrophages were calculated. The top 1 correlated module of high M2 macrophage infiltration was chosen for subsequent analyses. In the end, M2‐TAM genes were acquired by the intersection of modular genes and the TAM marker genes.

### Functional analysis

Gene ontology (GO, http://www.geneontology.org/) functional and Kyoto Encyclopedia of Genes and Genomes (KEGG, http://www.kegg.jp/kegg/pathway.html) pathway analyses were performed to evaluate the functions of the M2‐like TAM‐related genes by the clusterProfiler in R.^27^, ^28^, ^29^, ^30^

### Cluster analysis

The ConsensusClusterPlus was applied to implement cluster analysis to find M2‐like TAM‐related molecule subtypes. Then, the prognosis among the five clusters was analyzed using the Kaplan‐Meier method. The chi‐square test was performed to analyze the correlation between clusters and clinical features, and the results were visualized by the ggplot2 package in R. The immune, stromal, and estimate scores of samples were obtained from ESTIMATE (https://bioinformatics.mdanderson.org/estimate/), and differences of scores among the clusters were shown by ggpubr. Next, we estimated the immune cell infiltration of the five clusters by the CIBERSORT algorithm, and the differences among the clusters were visualized by the ggpubr and ggplot2 packages. Furthermore, Kaplan‐Meier analysis was performed to compare the survival of different immune cell infiltration extents. Immune checkpoint‐related transcripts and expression values were obtained from TCGA‐LUAD data. Significant differences in immune checkpoint genes among the five clusters were compared by the Kruskal–Wallis test.

### M2‐TAM genes based prognostic signature construction

First, M2‐TAM genes related to EGFR‐negative LUAD prognosis were found by univariate Cox (UniCox) analysis. Genes with *p* < 0.05 were regarded as prognostic genes. Then, least absolute shrinkage and selection operator (LASSO) regression analysis was conducted on M2‐like TAM prognostic genes. The model equation was as follows: Risk score = $\sum_{i=1}^{n}\mathrm{Ci}$ × *Expi*. The coefficients were produced by LASSO analysis. The signature accuracy was tested by receiver‐operating characteristic (ROC) curve analysis. High‐ and low‐risk groups were generated based on the median risk score. Kaplan‐Meier analysis was used to examine survival between the high‐ and low‐risk groups. The rms was employed to integrate survival time, survival status, T stage, TNM stage, age, sex, and the risk score. We assessed the prognostic significance of these features in TCGA samples and checked the congruence between the real data and the projected OS probability by calibration curves. Immunophenoscore (IPS) is a machine‐learning‐based system that calculates *z*‐scores based on four immunogenicity‐related cell types.^31^ For tumor mutational burden (TMB) estimation in EGFR‐negative LUAD, maftools was applied to calculate the mutation rate of each sample. The IPS and TMB between the high‐ and low‐risk groups was compared by the Wilcoxon rank sum test.

## RESULTS

### TAM marker genes of scRNA‐seq data

**FIGURE 2:**
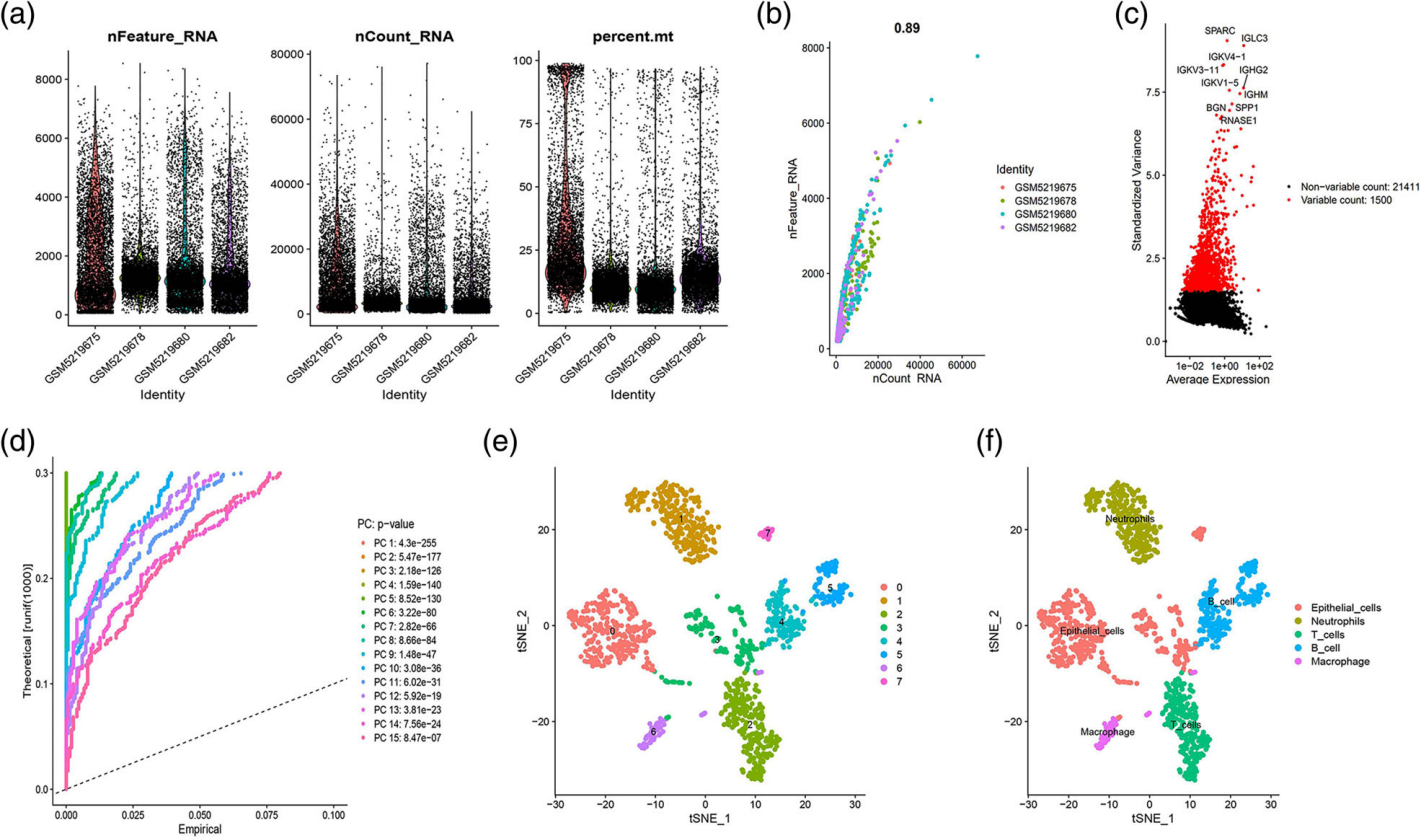
Identification of five cell clusters with diverse annotations revealing high cellular heterogeneity in EGFR‐negative lung adenocarcinoma (LUAD) based on scRNA‐seq data. (a) After quality control of scRNA‐seq, 1325 core cells were identified. (b) The number of genes detected was positively associated with the depth of sequencing. (c) The variance diagram shows the variation in gene expression in all cells of EGFR‐negative LUAD. The red dots represent highly variable genes, and the black dots represent nonvariable genes. (d) Principal component analysis (PCA) was used to identify the top 15 principal components (PCs) at *p* < 0.05. (e) The t‐SNE algorithm was applied to the top 15 PCs for dimensionality reduction. (f) Five cell clusters were successfully classified.

Figure 1 describes this study process in its entirety. First, we filtered low‐quality cells and identified 1325 high‐quality cells for subsequent analysis. The number of genes (nFeature), the sequence count per cell (nCount), and the percentage of mitochondrial genes (percent.mt) are shown in violin plots (Figure 2a). As shown in Figure 2b, nCount correlated positively with nFeature. Next, 1500 variable genes were plotted in a scatter diagram (Figure 2c). We also selected the top 15 PCs for t‐SNE analyses (Figure 2d). We conducted PCA using the 1500 variable genes to reduce the dimensionality, and eight cell clusters were then identified (Figure 2e). The FindAllMarkers function in the Seurat package was used to calculate the differentially expressed genes (DEGs) of each cluster using Wilcoxon‐Mann‐Whitney tests. To identify the marker genes for each cell cluster, the cutoff threshold values, adjusted *p*‐value <0.01 and |log2(fold change)| >1 were used. For cell cluster annotation, we performed a reference‐based annotation using reference data from the Human Primary Cell Atlas, and cells in cluster 6 were defined as macrophage cells (Figure 2f). The 199 marker genes of macrophages were selected (Table S1).

### Identification of M2 macrophage‐related genes by WGCNA

**FIGURE 3:**
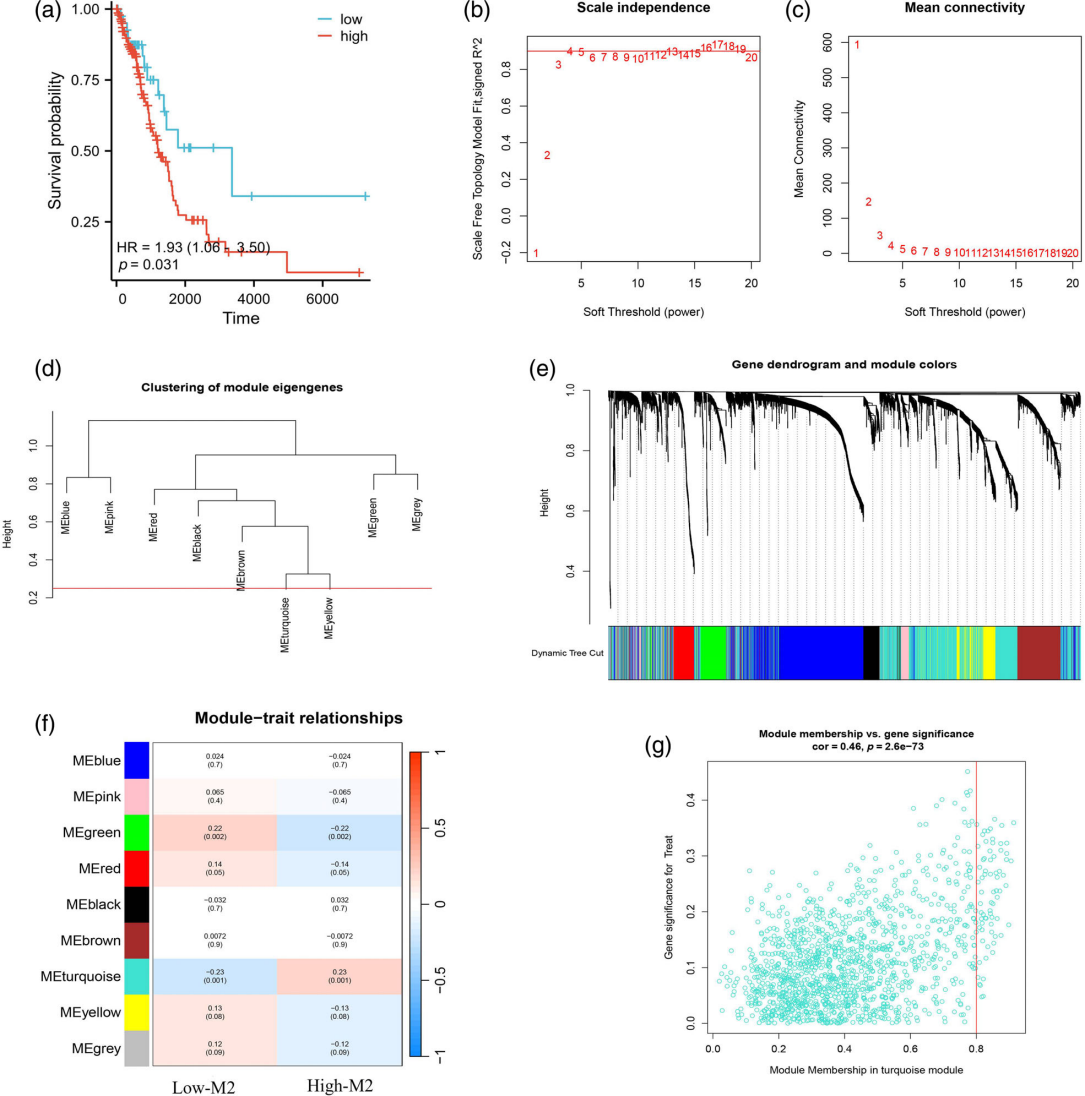
Macrophage‐related survival analysis and screening of M2 macrophage‐related genes. (a) Prognosis was significantly worse in the group with a high content of M2 macrophages. (b, c) According to the instructions of the WGCNA package, 4 was selected as the soft threshold power. (d) The minimum number of genes per module is 60, and nine modules are obtained when MEDissThres is equal to 0.25. (e, f) Correlation analysis of modules with traits yielded nine modules, with the turquoise module considered the most relevant module for M2 macrophages. (g) Scatter plot analysis of the turquoise module.

The TCGA data involved a total of 191 EGFR‐negative LUAD patients (82 men [42.93%] and 109 female [57.07%]; mean [SD] age, 65.76 [9.62] years; 171 individuals in T1–T2 [89.53%] and 20 in T3–T4 [10.74%]; 154 individuals were stage I– II [80.63%] and 34 individuals were stage III–IV [17.80%]; 162 individuals had a smoking history [84.82%], 28 individuals had no smoking history [14.66%]). The content of M2 macrophages in each TCGA‐EGFR‐negative LUAD sample was calculated on CIBERSORTx (https://cibersortx.stanford.edu/) using the default signature matrix. The X‐tile was used to calculate the best cutoff value to distinguish high‐ and low‐content groups of M2 macrophages in TCGA‐EGFR‐negative LUAD samples. The T and M stages showed a significant difference between the high‐ and low‐M2 macrophage content groups (*p* < 0.05, Table S2). Kaplan‐Meier analysis showed significant survival differences between the high‐ and low‐M2 macrophage content groups (Figure 3a). Furthermore, WGCNA was performed to screen out M2 macrophage‐related genes in EGFR‐negative LUAD. The soft threshold power β was 4 when the fit index of the scale‐free topology reached 0.85 (Figure 3b,c). MEDissThres was set to 0.25 to merge similar modules, and a total of nine modules remained (Figure 3d,e). MEturquoise with the highest correlation was regarded as the key module (including 1382 M2 macrophage‐related genes) (Figure 3f). The M2 macrophage‐related genes are listed in Table S3, and Figure 3g depicts the scatter plot of the turquoise module with clinical correlation.

### Functional and cluster analysis of M2‐TAMs genes

**FIGURE 4:**
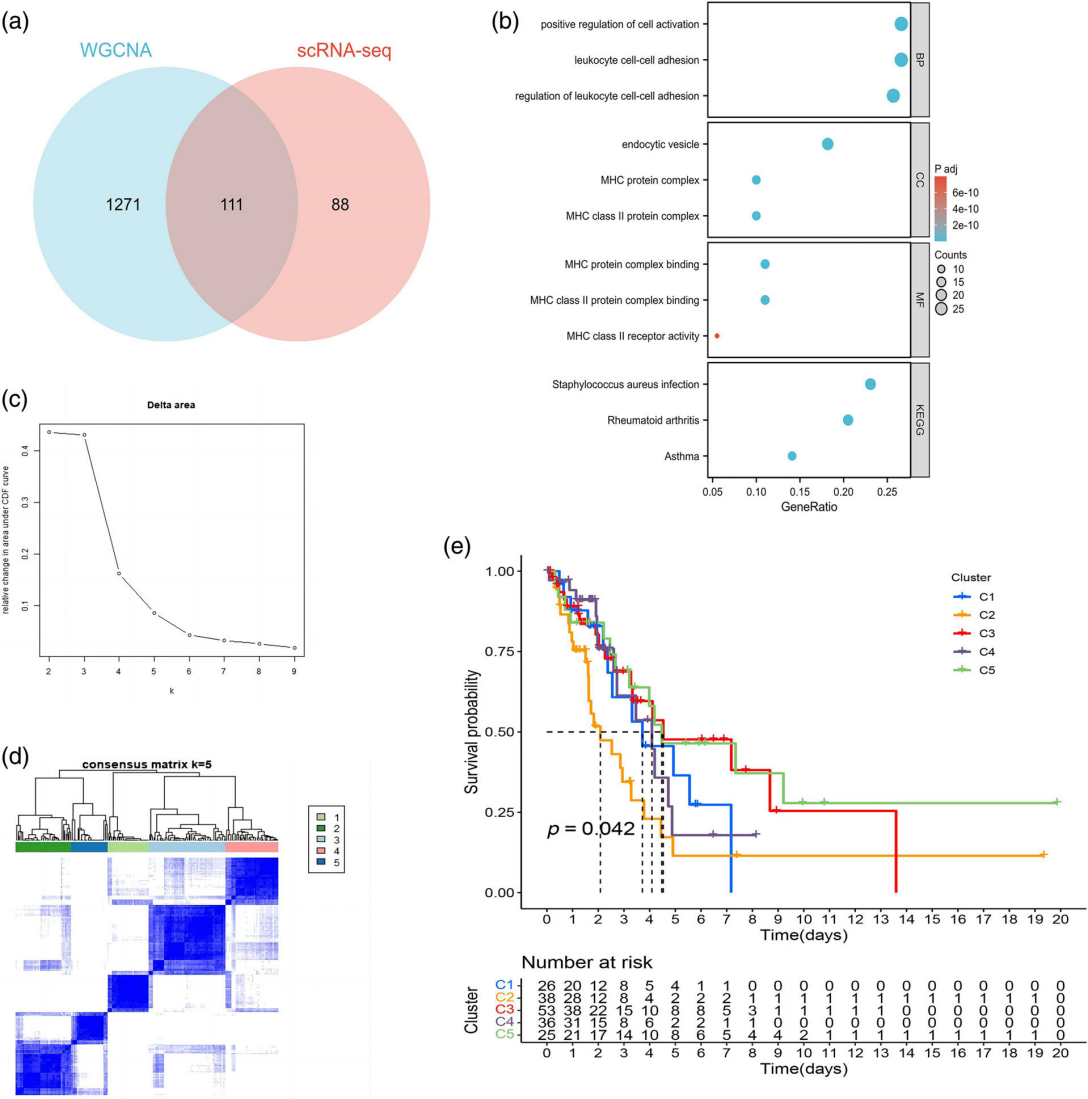
Identification of M2‐like tumor‐associated macrophage (TAM)‐related prognostic genes and unsupervised consensus clustering. (a) Screening out 111 M2‐like TAM‐related genes. (b) Gene ontology (GO) and Kyoto Encyclopedia of Genes and Genomes (KEGG) analyses of M2‐like TAM‐related prognostic genes. (c, d) Consensus clustering plot showing five to be a suitable *k* value, and EGFR‐negative lung adenocarcinoma (LUAD) samples were grouped into five clusters. (e) Kaplan‐Meier survival analysis was used to compare the differences in survival between the five clusters.

We identified 111 M2‐TAM genes through the intersection of the TAM marker genes and M2 macrophage‐related genes of WGCNA (Figure 4a). GO and KEGG analyses demonstrated that M2‐like TAM‐related genes were mainly enriched in the positive regulation of cell activation, endocytic vesicle, MHC protein complex binding, and *Staphylococcus aureus* infection (Figure 4b).

The area under the CDF curve increased significantly when k ≤5 (Figure 4c). Thus, EGFR‐negative TCGA‐LUAD samples were clustered into five clusters (Figure 4d). As shown in Figure 4e, survival among the five clusters exhibited obvious differences (*p* = 0.042), which indicated that these M2‐like TAM‐related genes have potential in prognosis prediction. However, we compared the differences of clinicopathological characteristics among the five clusters by Chi‐square test and there were no significant differences (*p* > 0.05, Figure S1).

**FIGURE 5:**
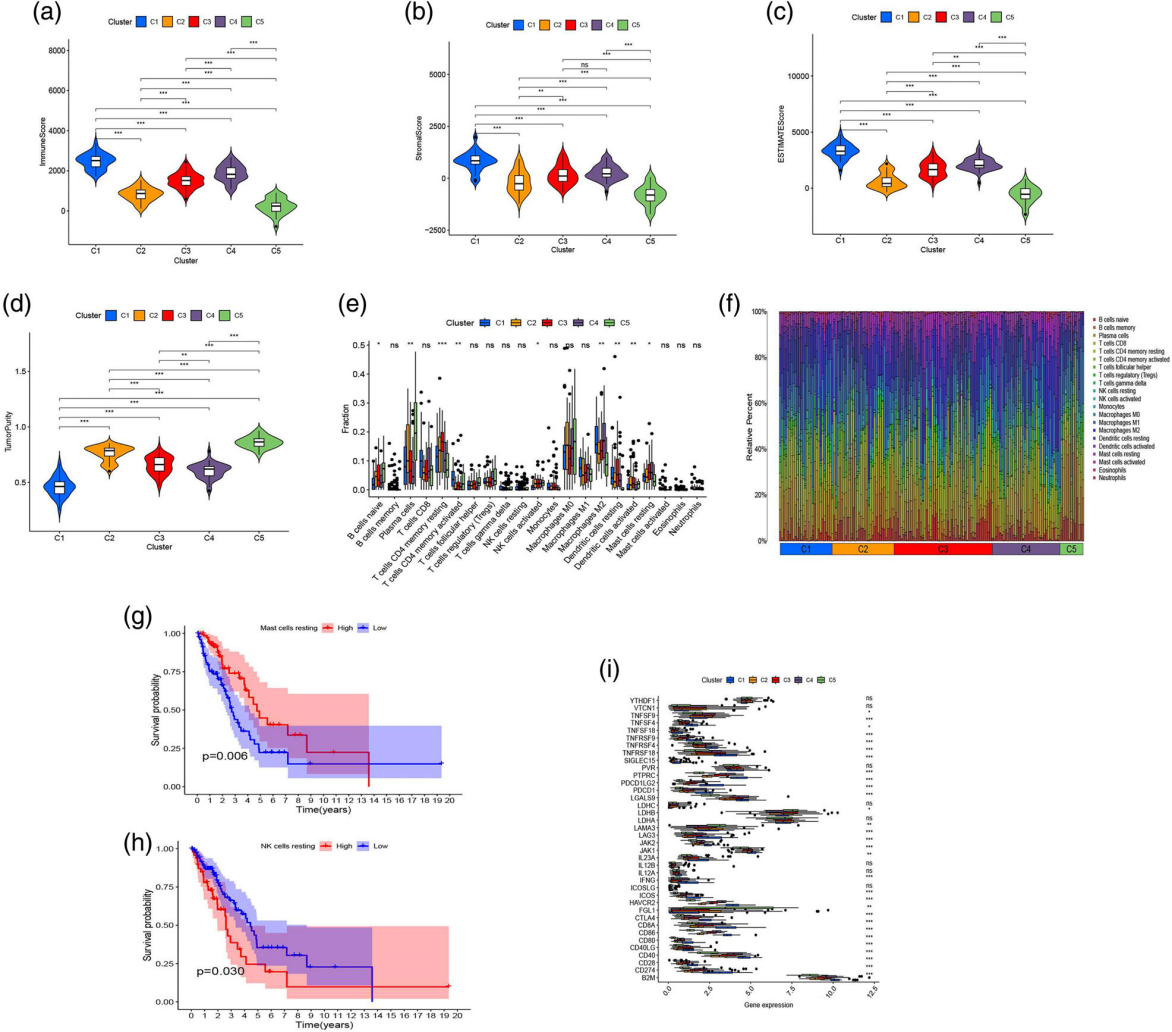
Differences in immune cells and immune function between the five clusters. (a–d) Differences in ImmuneScore, StromalScore, ESTIMATEScore and TumorPurity between the five clusters. (e) Comparison of immune cell content between the five clusters. (f) The percent of immune cells between the five clusters. (g) Kaplan‐Meier analysis was used to compare the survival differences between the high‐ and low‐resting mast cell group. (h) Kaplan Meier analysis was used to compare the survival difference between the high‐ and low‐resting NK cell group. (i) Differential expression of immune checkpoint genes between the five clusters. *p < 0.05, **p < 0.01, ***p < 0.001, ns, not significant.

Furthermore, differences in immune cells and immune function among the five clusters were analyzed. We observed significant differences in ImmuneScore, StromalScore, ESTIMATEScore and TumorPurity between the five clusters (Figure 5a–d). The contents of naive B cells, plasma cells, resting memory CD4 T cells, activated memory CD4 T cells, activated NK cells, M2 macrophages, resting dendritic cells, activated dendritic cells and resting mast cells showed significant differences between the five clusters (Figure 5e). Figure 5f presents the relative percentages of immune cells in the five clusters. As shown in Figure 4, the cluster 5 had better prognosis than other clusters, combined with the results in Figure 5, the cluster 5 had the lowest ImmuneScore, StromalScore and ESTIMATEScore, which may be related to poor prognosis. However, the TumorPurity was higher than other clusters, which indicated the TumorPurity related to better prognosis. As for the immune cell content of the cluster 5, the naive B cells, plasma cells, activated NK cells, and activated dendritic cells were the highest and suggested better prognosis; however, the resting memory CD4 T cells or M2 were the lowest in cluster 5 which related to poor prognosis. In addition, Kaplan‐Meier analysis showed that patients with a high abundance of resting mast cells had better prognosis (*p =* 0.006, Figure 5g) but that patients with a high abundance of resting NK cells had poor prognosis (*p* = 0.03, Figure 5h). In terms of the level of the immune checkpoints, many differentially expressed immune checkpoint genes between the five clusters were identified (Figure 5i), such as TNFSF4, CTLA4, CD40, and CD28. These results provide insight into discovering new targets for immunotherapy.

### Development of the M2‐TAM genes prognostic risk signature

**FIGURE 6:**
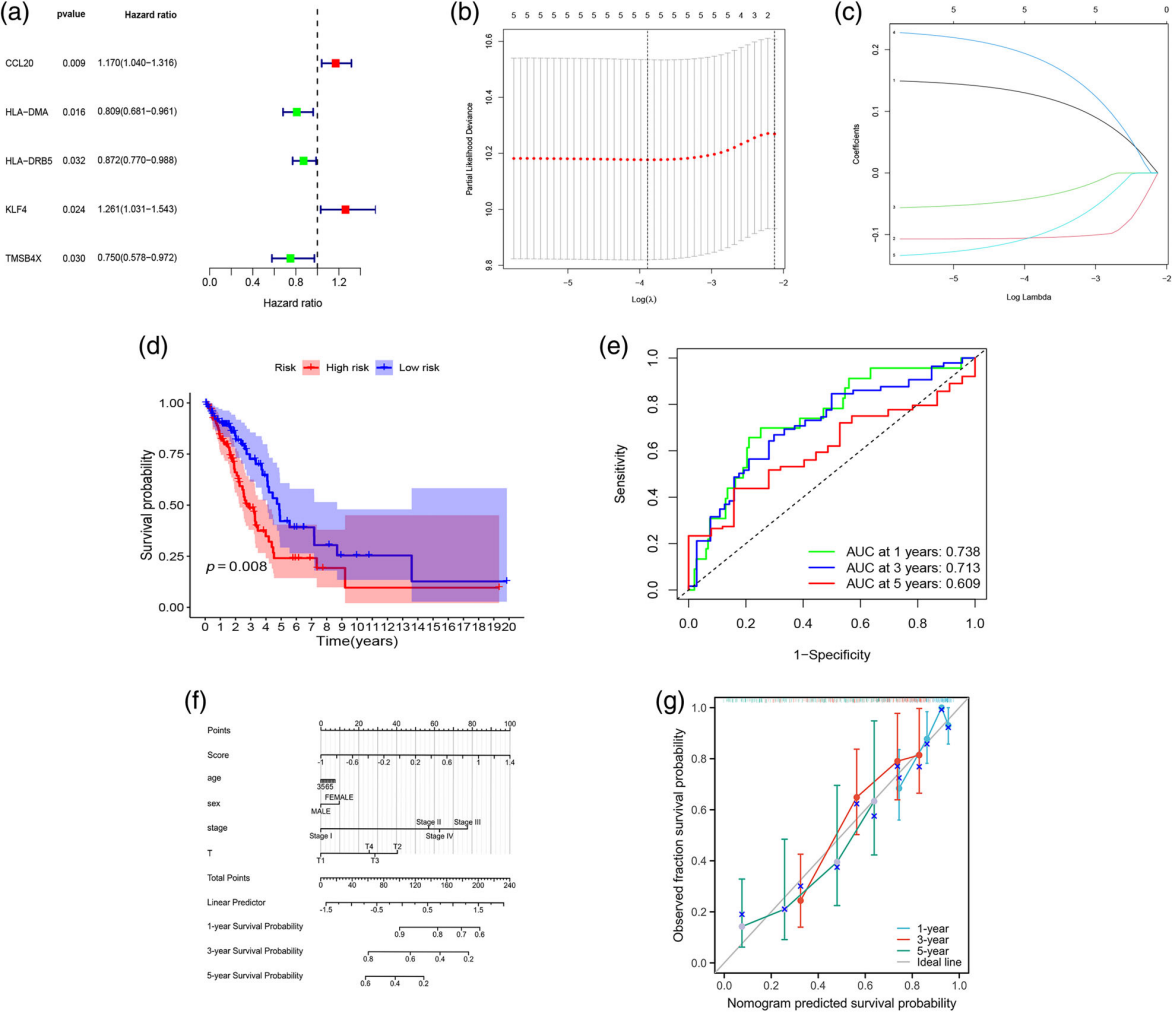
Prognostic model construction. (a) Forest map depicting the hazard ratio and *p*‐value derived from univariate Cox regression analysis. (b, c) LASSO regression of M2‐like tumor‐associated macrophage (TAM)‐related genes. (d) Kaplan‐Meier curve result. (e) Area under the curves (AUCs) of the prediction of 1‐, 3‐, and 5‐year survival rates of EGFR‐negative lung adenocarcinoma (LUAD). (f) Nomogram based on risk scores and clinical features. (g) Results of the calibration curve.

Univariate Cox regression analysis was conducted to screen potential prognostic genes from M2‐TAM genes with *p* < 0.05 (Figure 6a). Then, LASSO analysis identified five prognostic signature genes: CCL20, HLA‐DMA, HLA‐DRB5, KLF4, and TMSB4X (Figure 6b,c). Based on these five genes, a prognostic model was developed: Risk score = (0.127 × exp(CCL20)) + (−0.105 × exp(HLA‐DMA)) + (−0.041 × exp(HLA‐DRB5)) + (0.189 × exp (KLF4)) + (−0.104 × exp (TMSB4X)). To test the credibility of the prognostic signature, EGFR‐negative TCGA‐LUAD patients were grouped into high‐ and low‐risk groups according to the median risk score. In Figure 6d,e, patients in the low‐risk group showed better prognosis than those in the high‐risk group, with AUCs of 0.738, 0.713, and 0.609 for 1, 3, and 5 years, respectively. Stage, age, T stage, sex, and risk score were included in the nomogram (Figure 6f), and the calibration curve verified that the model may exert high predictive effect (Figure 6g).

**FIGURE 7:**
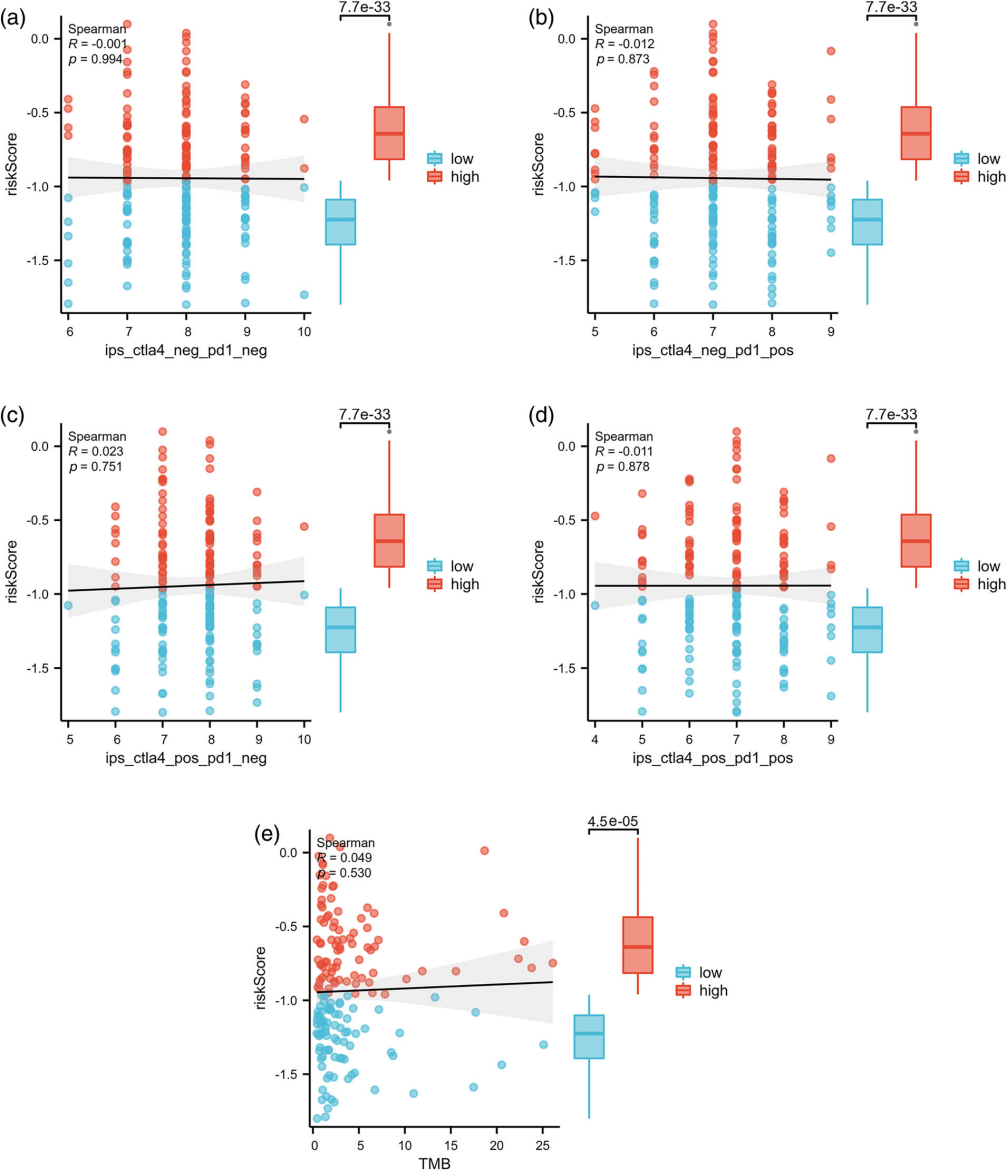
The correlation and comparison between riskScore and immunophenoscore (IPS)/tumor mutational burden (TMB). (a–d) Correlations and differences in the IPS between the two risk groups stratified by CTLA4 and PD‐1. (e) Correlations and differences in the TMB between the two risk groups.

Furthermore, the susceptibility of EGFR negative LUAD patients to ICIs was further assessed by using IPS. The results showed that the high‐risk group had higher IPS in any CTLA4 and PD‐L1 stratification than the low‐risk group, indicating that the relative probabilities of responding to ICIs in the high‐risk group were higher than those in the low‐risk group (Figure 7a–d). In addition, the high‐risk group had a higher TMB level (Figure 7e).

## DISCUSSION

Although LUAD harboring *EGFR* mutations prevails in the Asian population, many LUAD patients are without EGFR‐targeted therapeutic options, and the prognosis of EGFR‐negative patients is worse than that of patients harboring *EGFR* mutations. With the rapid progress in next‐generation sequencing, an increasing number of prognostic signatures based on transcriptome data to delineate individual differences are constantly emerging to predict prognosis in LUAD.^32^, ^33^, ^34^ However, few prognostic signatures have concentrated on EGFR‐negative LUAD with dismal prognosis. Thus, EGFR‐negative LUAD patients need a more credible prognostic model based on genetic changes to guide personalized treatment by early detection.

Macrophages are regarded as the most abundant non‐neoplastic cell types in the TME and primarily polarize into the M2‐like phenotype to promote immunosuppressive, anti‐inflammatory and angiogenic functions. M2 macrophages play an important role in LUAD tumor progression by producing Th2 cytokines.^35^ Because of the important position of M2‐TAMs in LUAD, there is increasing interest in M2‐TAMs‐based therapeutic approaches. For example, Lu et al. reported that targeting the Oct4/M‐CSF axis, which regulates M2 macrophage polarization, may be a new therapeutic method for lung cancer.^36^ HHLA2 has been reported as a potential therapeutic target that inhibits TAM M2 polarization in NSCLC.^37^ In the current study, we first found that M2‐like TAMs were associated with decreased survival in patients with EGFR‐negative LUAD. Next, EGFR‐negative LUAD scRNA‐seq data were analyzed, and an innovative prognostic signature was constructed based on five M2‐like TAM‐related genes, including CCL20, HLA‐DMA, HLA‐DRB5, KLF4, and TMSB4X. The functions of these five genes have been reported in several previous articles. CCL20 plays an important role in remodeling of the TME.^38^, ^39^ Furthermore, Xu et al. found that the miR‐1322/CCL20 axis may regulate M2 polarization in colorectal cancer and provided new targeting chemokines for cancer therapy.^40^ HLA‐DMA and HLA‐DRB5 are part of the major histocompatibility complex (MHC) II molecules, which are mainly present on professional antigen‐presenting cells.^41^ Morafraile et al. previously observed that the well‐known immune‐related genes HLA‐DMA and HLA‐DRB5 correlate positively with macrophages in colon adenocarcinoma.^42^ Prior studies have also reported that KLF4 induces M2 polarization,^43^, ^44^ which is consistent with our findings. TMSB4X was found to be overexpressed in malignant and metastatic cells by analyzing the single‐cell transcriptome of papillary thyroid cancer, which indicates that it may participate in cancer progression.^45^ All these studies showed the importance of the five M2‐TAMs‐genes in tumor progression, and the creative prognostic signature in our study achieved good performance in prediction. Compared with another analogous model,^46^ the signature in our study synthesizes scRNA‐seq and TCGA datasets by multiple algorithms, and the AUC values of the signature reached 0.609–0.738, indicating high reliability and relevance. In addition, the signature may be an indicator of immunotherapy response. To support this hypothesis, we found that the high‐risk group had a higher TMB, which indicated that patients with a high‐risk score were more likely to benefit from immunotherapy.

Finally, developing predictive gene signature for ICI treatment has always been important for screening treatment populations to achieve precise treatment. Many predictive biomarkers of ICI therapy have been developed, such as PD‐L1 expression and CD8 infiltration.^47^, ^48^ In our study on M2‐TAM‐related gene signature to evaluate the treatment response to ICIs in the two risk groups, we found that high‐risk patients had more antitumor immune infiltrating cells and a better immunotherapy response. This might impact the survival and prognosis of EGFR‐negative LUAD patients, and the five M2‐TAM‐related gene signature might act as a biomarker for ICI therapy in EGFR‐negative LUAD patients.

In summary, the advantage of this study lies in our construction of a novel M2‐TAM‐prognostic signature capable of accurately distinguishing OS outcomes and immunotherapy responses, and the drug prediction results will be of interest for precise treatment of EGFR‐negative LUAD patients. However, some limitations should be noted, including the small sample size and the fact that the regulatory mechanisms of signature genes remain unclear. Further studies are needed to address these issues.

In conclusion, this study involved multiple methods to establish a novel M2‐TAM‐prognostic signature for OS prediction in EGFR‐negative LUAD patients. This signature provides new insight into drug targets and immunotherapy response prediction.

## AUTHOR CONTRIBUTIONS

Conceptualization: Yajie Huang and Jian Shi. Methodology: Qi Wang. Software: Yajie Huang. Validation: Xiaoyang Duan, Ran Hou, and Jian Shi. Investigation: Yajie Huang. Resources: Qi Wang. Data curation: Yajie Huang. Writing—original draft preparation: Yajie Huang. Writing‐review and editing: Yaozhong Zhang. Visualization: Ran Hou. Supervision: Jian Shi. Project administration: Yajie Huang. Funding acquisition: Yajie Huang. All the authors have read and approved the final manuscript.

## CONFLICT OF INTEREST STATEMENT

The authors declare that they have no competing interests.

## Supporting information


**Data S1:** Supporting Information.

## Data Availability

The datasets generated and/or analyzed during the current study are available at the TCGA (https://portal.gdc.cancer.gov/projects/TCGA) and GEO (accession number: GSE171145) (https://www.ncbi.nlm.nih.gov/geo/). All original data generated in the present study can be directed to Yajie Huang (2679588968@qq.com).
