# Supplementary material for: Exploring the immune landscape and drug prediction of an M2 tumor‐associated macrophage‐related gene signature in EGFR‐negative lung adenocarcinoma
Source: Thorac Cancer. 2024 Jun 17;15(21):1626–37. doi: 10.1111/1759-7714.15375 (PMC11260554; doi:10.1111/1759-7714.15375)
Supplement: Supplementary file 1 — Data S1: Supporting Information. [file TCA-15-1626-s001.docx]

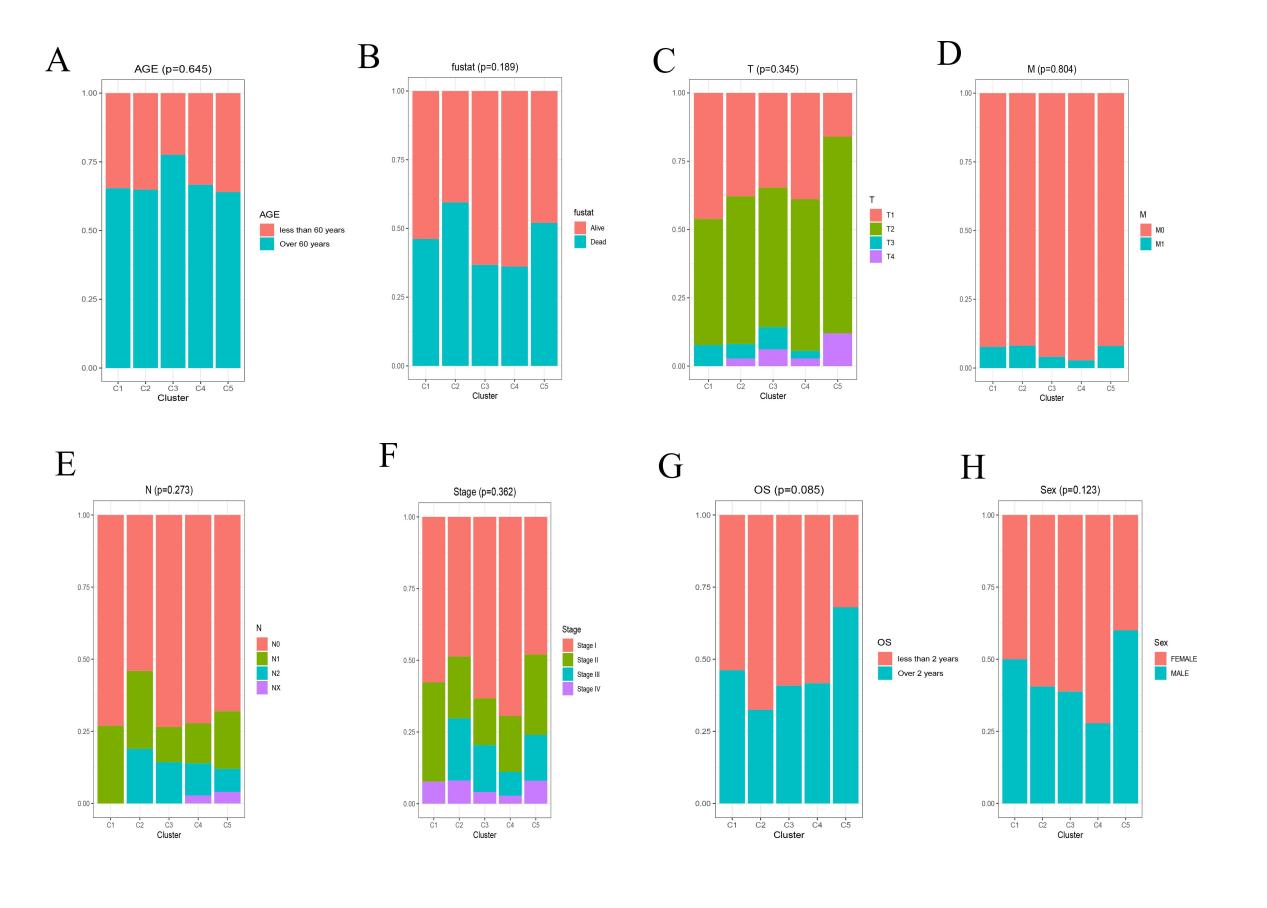


Fig. S1 Histograms of clinicopathological features. (A) Age. (B) Fustat. (C) Tumor size. (D) Metastasize. (E) Lymph node. (F) Stage. (G) OS status. (H) Sex.

Table S1 TAMs marker genes of Macrophage cells

| Macrophage | DAB2 |
| --- | --- |
| Macrophage | MS4A7 |
| Macrophage | FPR3 |
| Macrophage | SDS |
| Macrophage | MSR1 |
| Macrophage | C1QC |
| Macrophage | MS4A6A |
| Macrophage | LILRB4 |
| Macrophage | APOE |
| Macrophage | CD86 |
| Macrophage | VSIG4 |
| Macrophage | MS4A4A |
| Macrophage | C1QA |
| Macrophage | LYZ |
| Macrophage | IL4I1 |
| Macrophage | SIGLEC1 |
| Macrophage | FCGR3A |
| Macrophage | ADAP2 |
| Macrophage | C1QB |
| Macrophage | APOC1 |
| Macrophage | RASSF4 |
| Macrophage | CSF1R |
| Macrophage | GPNMB |
| Macrophage | IRF8 |
| Macrophage | SLCO2B1 |
| Macrophage | HMOX1 |
| Macrophage | KCTD12 |
| Macrophage | CAPG |
| Macrophage | PMP22 |
| Macrophage | ZNF385A |
| Macrophage | CFD |
| Macrophage | TGFBI |
| Macrophage | GNB4 |
| Macrophage | MPEG1 |
| Macrophage | SLAMF8 |
| Macrophage | PLXDC2 |
| Macrophage | AXL |
| Macrophage | CTSB |
| Macrophage | C1orf162 |
| Macrophage | HLA-DPA1 |
| Macrophage | CCDC88A |
| Macrophage | RNF130 |
| Macrophage | NRP1 |
| Macrophage | GM2A |
| Macrophage | CTSZ |
| Macrophage | LY86 |
| Macrophage | HLA-DPB1 |
| Macrophage | CD163 |
| Macrophage | HLA-DQA2 |
| Macrophage | TTYH3 |
| Macrophage | SPP1 |
| Macrophage | FCER1G |
| Macrophage | CTSL |
| Macrophage | MAFB |
| Macrophage | MFSD1 |
| Macrophage | TYROBP |
| Macrophage | TFEC |
| Macrophage | NPL |
| Macrophage | HLA-DRA |
| Macrophage | LAIR1 |
| Macrophage | TMEM176B |
| Macrophage | RNASE6 |
| Macrophage | CTSD |
| Macrophage | SIRPA |
| Macrophage | CD74 |
| Macrophage | SLC7A7 |
| Macrophage | RAB31 |
| Macrophage | GRN |
| Macrophage | GPX1 |
| Macrophage | AIF1 |
| Macrophage | FTL |
| Macrophage | FTLP3 |
| Macrophage | PSAP |
| Macrophage | ACP5 |
| Macrophage | RAB20 |
| Macrophage | HLA-DQB1 |
| Macrophage | CREG1 |
| Macrophage | TIMP2 |
| Macrophage | A2M |
| Macrophage | MRC1 |
| Macrophage | CCL2 |
| Macrophage | SLC15A3 |
| Macrophage | CD14 |
| Macrophage | PLD3 |
| Macrophage | ARHGAP18 |
| Macrophage | SLC2A5 |
| Macrophage | HLA-DRB1 |
| Macrophage | CYBB |
| Macrophage | FCGRT |
| Macrophage | TMEM176A |
| Macrophage | HNMT |
| Macrophage | SERPING1 |
| Macrophage | LIPA |
| Macrophage | FCGR2A |
| Macrophage | CXCL3 |
| Macrophage | KLF4 |
| Macrophage | VIM |
| Macrophage | LGALS3 |
| Macrophage | LGALS1 |
| Macrophage | LGMN |
| Macrophage | HLA-DRB6 |
| Macrophage | VAT1 |
| Macrophage | SDC3 |
| Macrophage | MPP1 |
| Macrophage | IL2RA |
| Macrophage | PEA15 |
| Macrophage | LAPTM5 |
| Macrophage | CTSH |
| Macrophage | HLA-DMB |
| Macrophage | FMNL2 |
| Macrophage | HK2 |
| Macrophage | LGALS9 |
| Macrophage | ABCA1 |
| Macrophage | HLA-DRB5 |
| Macrophage | THBD |
| Macrophage | DNASE2 |
| Macrophage | HEXB |
| Macrophage | HLA-DQA1 |
| Macrophage | MMP14 |
| Macrophage | BCAT1 |
| Macrophage | TFRC |
| Macrophage | GPR183 |
| Macrophage | ANXA2 |
| Macrophage | RNASE1 |
| Macrophage | GSN |
| Macrophage | CST3 |
| Macrophage | ATP6V1F |
| Macrophage | INSIG1 |
| Macrophage | NUPR1 |
| Macrophage | MT2A |
| Macrophage | CTSC |
| Macrophage | TMSB4X |
| Macrophage | CD4 |
| Macrophage | YWHAH |
| Macrophage | CCL18 |
| Macrophage | COLGALT1 |
| Macrophage | OTUD1 |
| Macrophage | RNASET2 |
| Macrophage | RGS10 |
| Macrophage | GADD45B |
| Macrophage | DBI |
| Macrophage | RB1 |
| Macrophage | CORO1C |
| Macrophage | PLTP |
| Macrophage | ITGB2 |
| Macrophage | HBEGF |
| Macrophage | VEGFB |
| Macrophage | GRB2 |
| Macrophage | TPP1 |
| Macrophage | CD81 |
| Macrophage | FCGR2B |
| Macrophage | SLC29A1 |
| Macrophage | PARVG |
| Macrophage | ADA2 |
| Macrophage | AKR1B1 |
| Macrophage | NPC2 |
| Macrophage | GLUL |
| Macrophage | AP1B1 |
| Macrophage | ADM |
| Macrophage | EMP3 |
| Macrophage | RASGEF1B |
| Macrophage | IFNGR2 |
| Macrophage | PLEK |
| Macrophage | BRI3 |
| Macrophage | ABHD12 |
| Macrophage | GPI |
| Macrophage | IER5 |
| Macrophage | SCARB2 |
| Macrophage | SAMHD1 |
| Macrophage | CXCL2 |
| Macrophage | GNAI2 |
| Macrophage | IFI44L |
| Macrophage | CSTB |
| Macrophage | CD63 |
| Macrophage | DPYSL2 |
| Macrophage | CXCL16 |

Table S2. The Comparison of Clinical Characteristics between High- and Low-M2 Group

| Characteristics | High-M2 | Low-M2 | P value |
| --- | --- | --- | --- |
| n | 147 | 44 |  |
| T.stage, n (%) |  |  | 0.035 |
| T1 | 43 (22.5%) | 22 (11.5%) |  |
| T2 | 86 (45%) | 20 (10.5%) |  |
| T3 | 11 (5.8%) | 0 (0%) |  |
| T4 | 7 (3.7%) | 2 (1%) |  |
| N.stage, n (%) |  |  | 0.587 |
| N0 | 100 (52.4%) | 33 (17.3%) |  |
| NX | 1 (0.5%) | 1 (0.5%) |  |
| N1 | 29 (15.2%) | 6 (3.1%) |  |
| N2 | 17 (8.9%) | 4 (2.1%) |  |
| M.stage, n (%) |  |  | 0.043 |
| M0 | 108 (56.5%) | 29 (15.2%) |  |
| MX | 29 (15.2%) | 15 (7.9%) |  |
| M1 | 10 (5.2%) | 0 (0%) |  |
| stage, n (%) |  |  | 0.296 |
| Stage I | 82 (43.6%) | 29 (15.4%) |  |
| Stage II | 34 (18.1%) | 9 (4.8%) |  |
| Stage III | 18 (9.6%) | 6 (3.2%) |  |
| Stage IV | 10 (5.3%) | 0 (0%) |  |
| Age, median (IQR) | 67 (59, 73) | 63.5 (58.75, 71.25) | 0.216 |
| Smoking history, n (%) |  |  | 0.869 |
| No | 22 (11.6%) | 6 (3.2%) |  |
| Yes | 125 (65.8%) | 37 (19.5%) |  |
| Gender, n (%) |  |  | 0.177 |
| FEMALE | 80 (41.9%) | 29 (15.2%) |  |
| MALE | 67 (35.1%) | 15 (7.9%) |  |

Table S2. The M2 macrophage-related genes

AASS

ABCA1

ABCA12

ABCA4

ABCG1

ABHD17C

ABI3

ABLIM1

ABLIM3

AC002316.1

AC004151.1

ACAD8

ACE

ACMSD

ACP5

ACSL1

ADA2

ADAM15

ADAM8

ADAMTSL4

ADD3

ADGRE5

ADGRF1

ADGRG1

ADK

ADORA1

ADORA2B

ADPRHL1

ADRA2C

ADSS1

AFAP1L2

AGA

AHNAK

AHR

AIF1

AIM2

AKR1B1

AKR7A3

ALDH1A3

ALDH3A2

ALDOC

ALG1L

ALOX5

ALOX5AP

ALPP

AMBP

AMIGO2

AMN

AMOT

ANKRD22

ANKRD9

ANOS1

ANPEP

ANTXR2

ANXA1

ANXA13

ANXA2

ANXA3

AOAH

APBB1IP

APLP1

APOBEC2

APOBEC3C

APOBR

APOC1

APOE

APOH

APOL4

AQP1

AQP9

AREG

ARFGEF3

ARG2

ARHGAP29

ARHGAP30

ARHGAP31

ARHGDIB

ARHGEF26

ARHGEF4

ARMCX1

ARMCX2

ARRDC4

ARSJ

ASB9

ASCL1

ASPG

ASPHD1

ASRGL1

ATF3

ATP10A

ATP10B

ATP13A4

ATP1B1

ATP6V0A4

ATP6V0D2

ATP6V1B1

ATP7B

ATP8B1

AVPI1

AXL

AZGP1

B2M

B3GNT7

B4GALNT2

BAAT

BAG1

BARX1

BARX2

BASP1

BATF

BATF3

BCAS1

BCAT1

BCL2A1

BCL2L15

BDKRB2

BEX1

BEX2

BEX4

BHLHA15

BHLHE40

BHLHE41

BHMT2

BICDL1

BIK

BIN2

BIRC3

BIRC7

BMP4

BMP6

BNIP3

BPIFA2

BPIFB2

BPIFB4

BRDT

BRINP1

BSPRY

BST2

BTK

C16orf74

C19orf33

C1GALT1

C1orf115

C1orf162

C1orf210

C1orf226

C1QA

C1QB

C1QC

C3AR1

C4A

C4B

C5AR1

C6orf141

C6orf223

C6orf52

C8B

CA11

CA12

CA13

CA2

CA8

CA9

CACHD1

CACNA1H

CADM4

CALCA

CALML3

CAMK2D

CAPG

CAPN5

CAPN6

CARD11

CARD14

CARD16

CASP1

CATSPER1

CATSPERB

CAV1

CAV2

CBLC

CBX6

CCBE1

CCDC69

CCDC71L

CCDC85B

CCL13

CCL17

CCL18

CCL2

CCL22

CCL23

CCL24

CCL28

CCL3

CCL3L3

CCL4L2

CCL8

CCR1

CCRL2

CD14

CD163

CD1A

CD1B

CD1C

CD1E

CD207

CD209

CD24

CD274

CD300A

CD300C

CD300LF

CD4

CD40

CD44

CD47

CD48

CD52

CD53

CD69

CD74

CD82

CD83

CD84

CD86

CD9

CDC25B

CDC42EP1

CDC42EP3

CDCP1

CDH3

CDK2AP2

CDKN2B

CDR2L

CEACAM1

CEACAM4

CEBPB

CECR2

CELF3

CENPV

CENPX

CERKL

CES3

CFD

CFH

CFTR

CGA

CGN

CGREF1

CH25H

CHAC1

CHCHD10

CHGB

CHI3L1

CHIT1

CHL1

CHML

CHMP4C

CHP1

CHPT1

CHRD

CHRDL2

CHRNA5

CHST11

CHST2

CHST3

CIART

CIITA

CITED4

CKMT1B

CLCF1

CLDN1

CLDN10

CLDN4

CLDN7

CLEC10A

CLEC12A

CLEC2B

CLEC4A

CLEC5A

CLEC7A

CLIC2

CLPSL2

CLTRN

CMKLR1

CMPK2

CMTM3

CNGA3

CNN3

COL17A1

COL21A1

COL25A1

COL4A5

COLEC12

COTL1

CPA3

CPE

CPS1

CPVL

CRABP2

CREB3L4

CRYBG1

CSF1

CSF1R

CSF2

CSF2RA

CSF2RB

CSF3R

CSPG5

CST6

CSTA

CTAG2

CTNND2

CTSB

CTSC

CTSO

CTSS

CTSZ

CUEDC1

CX3CL1

CX3CR1

CXADR

CXCL14

CXCL2

CXCL3

CXXC4

CYBA

CYBB

CYP1B1

CYP24A1

CYP27A1

CYP27B1

CYP2S1

CYP7B1

CYTH3

CYTH4

DAB2

DACT2

DAPP1

DCBLD1

DCBLD2

DCDC2

DCXR

DDC

DDIT4

DDIT4L

DDO

DDX58

DDX60

DEFB1

DEFB4A

DHDH

DHRS9

DIO1

DIRAS3

DMBT1

DMKN

DNAJC12

DNAJC15

DOCK2

DOCK5

DOCK8

DOK2

DPM3

DPP4

DPY19L1

DPYD

DRGX

DTX2

DUSP10

DUSP23

DUSP4

DUSP6

EBPL

EDARADD

EDN2

EFEMP1

EFHD2

EFNA1

EFNA3

EFNB1

EGFR

EGR1

EGR2

EGR3

EHD2

EHF

EIF1AY

ELF3

ELFN2

ELK3

EMB

EMILIN2

EMP3

ENDOD1

ENG

ENO2

ENO3

ENPP2

ENPP3

ENTPD2

ENTPD8

EPB41L3

EPHA1

EPHA2

EPHB2

EPN3

EPSTI1

ERBB2

ERBB3

EREG

ERFE

ERICH5

ESR1

ETNK2

ETNPPL

ETS2

ETV1

ETV4

ETV5

EVA1A

EVI2A

EVI2B

EZR

F13A1

F2

F3

FA2H

FAAH2

FABP3

FABP5

FAM107B

FAM110C

FAM126A

FAM174B

FAM177A1

FAM20A

FAM3B

FAM50B

FAM89A

FAS

FASN

FAXDC2

FBXO6

FCER1G

FCGBP

FCGR1A

FCGR2A

FCGR2C

FCGR3A

FCN1

FERMT3

FGA

FGB

FGFBP1

FGG

FGL1

FGL2

FGR

FHOD3

FKBP2

FKBP5

FLNA

FN3K

FNIP2

FOLR2

FOSB

FOXA3

FOXC1

FOXI3

FOXQ1

FPR1

FPR3

FREM2

FST

FSTL4

FTL

FUOM

FURIN

FUT8

FXYD3

FXYD4

FXYD5

FZD10

G0S2

GAA

GABBR2

GADD45A

GADD45B

GAGE2A

GAL3ST1

GAL3ST4

GALNT10

GALNT5

GALNT7

GATM

GBP2

GBP3

GBP6

GCNT1

GDPD3

GGT1

GGT5

GIMAP2

GIMAP4

GIMAP6

GLB1L3

GLIPR1

GLIPR2

GLRX

GLS

GLTPD2

GMDS

GMFG

GNA15

GNAI1

GNG12

GOLM1

GP2

GPAT2

GPD1

GPNMB

GPR132

GPR137B

GPR143

GPR183

GPR27

GPR34

GPR68

GPR84

GPR87

GPRIN2

GPSM3

GPT2

GRAMD2A

GRASP

GRB14

GRB7

GREM2

GRTP1

GSDME

GSPT2

GSTA4

GSTP1

GUCA2B

GYG2

H1-2

H2AJ

H2AW

H2BC12

H2BC21

H2BC4

H4C8

H4C9

HACD1

HAL

HAVCR2

HBEGF

HCAR2

HCK

HCLS1

HCST

HELZ2

HERC5

HERC6

HES6

HEY1

HGSNAT

HHIP

HHLA2

HID1

HIF1A

HIF3A

HIP1

HK3

HLA-A

HLA-B

HLA-C

HLA-DMA

HLA-DMB

HLA-DOA

HLA-DPA1

HLA-DPB1

HLA-DQA1

HLA-DQA2

HLA-DQB1

HLA-DQB2

HLA-DRA

HLA-DRB1

HLA-DRB5

HLA-E

HLA-F

HLA-G

HMGB3

HMOX1

HORMAD1

HOXC10

HOXC11

HOXC13

HOXC9

HOXD1

HOXD9

HPGD

HPGDS

HPN

HS3ST1

HS3ST2

HS6ST2

HSD11B1

HSD17B14

HSPA6

HSPB1

HSPB8

ICAM1

ID1

ID2

ID4

IDS

IFFO2

IFI16

IFI27

IFI27L2

IFI35

IFI44

IFI44L

IFI6

IFIH1

IFIT1

IFIT2

IFIT3

IFITM1

IFITM10

IFITM3

IFT57

IGF1R

IGFBP6

IGSF6

IL10RA

IL13RA2

IL17RB

IL18

IL1B

IL1R1

IL1RAP

IL1RL1

IL1RN

IL23A

IL27RA

IL2RA

IL37

IL4I1

IL6

IMPA2

IMPDH1

INAVA

INHA

INSIG1

INSL4

IPCEF1

IQGAP2

IRAK2

IRAK3

IRF5

IRF7

IRF8

IRS2

ISG15

ISYNA1

ITGA2

ITGA3

ITGAM

ITGAX

ITGB2

ITGB3

ITGB8

ITIH2

ITLN1

ITPKA

ITPR3

IVL

IYD

JADE1

JAML

JUN

JUNB

KAZALD1

KCNA3

KCNAB2

KCNE4

KCNJ11

KCNJ5

KCNK5

KCNK6

KCNN4

KCNQ3

KCTD12

KCTD14

KCTD15

KCTD3

KIF13B

KIT

KITLG

KLB

KLF4

KLF5

KLF6

KLHDC7A

KLHDC9

KLHL35

KLK7

KRCC1

KRT13

KRT4

KRT7

KRTCAP3

KSR1

LAD1

LAIR1

LAMA3

LAMP3

LAPTM4B

LAPTM5

LARGE2

LAT2

LCN15

LCP1

LCP2

LDLR

LDOC1

LEMD1

LFNG

LGALS1

LGALS3

LGALS3BP

LGALS9

LGR4

LGSN

LHFPL2

LHFPL4

LILRA5

LILRB1

LILRB2

LILRB4

LIMCH1

LIMK1

LIPA

LIPH

LIX1L

LMO4

LPAR1

LPCAT1

LPXN

LRATD1

LRIG3

LRP11

LRP4

LRRC25

LRRC26

LRRC31

LRRN4

LSP1

LST1

LTK

LY6E

LY86

LY96

LYPD1

LYPD6B

LYZ

MACC1

MACROD1

MAEL

MAFB

MAFK

MAGEA1

MAGEA10

MAGEB2

MAGI3

MAML2

MAMLD1

MAN1A1

MANEAL

MANSC1

MAP1LC3C

MAP7D2

MAPK11

MAPK4

MARC1

MARCKS

MARCO

MARK1

MBOAT1

MBOAT2

MCEMP1

MCF2L

MCUB

MDFIC

MET

METRNL

METTL7B

MFSD4A

MICAL2

MICB

MID1IP1

MILR1

MIOX

MISP

MMP19

MMP24

MMP28

MMP7

MNDA

MPEG1

MPP1

MPP7

MRC1

MS4A4A

MS4A6A

MS4A7

MSLNL

MSMB

MSN

MSR1

MT1A

MT1E

MT1F

MT1G

MT1H

MT1M

MT1X

MT2A

MTMR7

MUC21

MUC4

MUC5AC

MX1

MX2

MYBPH

MYCL

MYCN

MYH14

MYO10

MYO1F

MYO1G

MYO6

MYOF

NAB2

NAGS

NAT8L

NAXD

NCEH1

NCF2

NCF4

NCKAP1L

NCMAP

NCOA7

NDP

NDUFB9

NEBL

NECAB3

NECTIN1

NECTIN3

NECTIN4

NEDD4L

NEFL

NFAM1

NFKBIA

NFKBIZ

NHS

NHSL1

NIBAN1

NKX2-8

NMB

NMNAT2

NOTUM

NPAS3

NPL

NPW

NR1D1

NR4A2

NRGN

NRK

NRN1

NRP1

NRP2

NT5E

NTN4

NTS

NUPR1

OAS1

OAS2

OAS3

OASL

OAT

OBP2A

OBSL1

OCIAD2

ODC1

OGFRL1

OLFML3

OLR1

OSBPL5

OSCAR

OSM

OSMR

OVGP1

OXCT1

P2RY13

PABPC1

PACSIN3

PADI1

PADI2

PADI3

PAG1

PAGE1

PAK3

PALD1

PAPSS2

PAQR8

PARD6A

PAX7

PBX1

PC

PCDHB11

PCED1B

PCOLCE2

PCSK1

PCSK1N

PCSK9

PDCD1LG2

PDE3A

PDE4D

PDIA3

PDLIM4

PDP1

PDX1

PDZK1IP1

PEG10

PELI1

PENK

PER3

PEX6

PFKFB3

PHLDA1

PHLDA2

PI3

PIEZO1

PIK3AP1

PIK3C2G

PIK3IP1

PILRA

PIM3

PITPNC1

PITX2

PKIA

PKP1

PLA1A

PLA2G4A

PLA2G4E

PLA2G7

PLAAT3

PLAAT4

PLAT

PLAU

PLBD1

PLCB2

PLEK

PLEKHA4

PLEKHA6

PLEKHF1

PLEKHG2

PLEKHO1

PLEKHO2

PLPP2

PLS3

PLSCR1

PLTP

PLXND1

PMP22

PNMA6A

PODXL2

PON2

PON3

PPARG

PPARGC1A

PPBP

PPL

PPP1R14C

PPP1R15A

PPP1R18

PPP1R3B

PRKACB

PRKCA

PRMT6

PRNP

PROC

PROCR

PROM2

PROSER2

PRR36

PRR5L

PRSS1

PRSS2

PRSS21

PRXL2B

PSMB10

PSMB8

PTAFR

PTGER2

PTGER4

PTP4A1

PTPN22

PTPRG

PYCARD

RAB11FIP1

RAB15

RAB20

RAB25

RAB26

RAB27B

RAB32

RAB37

RAB38

RAB42

RAB7B

RAC2

RAI14

RAMP1

RAPGEFL1

RASAL1

RASD1

RASGRF1

RASGRP1

RASL11A

RASSF10

RASSF2

RASSF4

RASSF5

RASSF8

RASSF9

RBP4

RBPMS2

RDH10

REG1A

RENBP

RET

RETN

RGL1

RGS1

RGS10

RGS17

RGS2

RHBDF2

RHBDL1

RHBDL2

RHOC

RIMKLB

RNASE2

RNASE6

RNF186

RNF19A

RNF213

RNF43

ROS1

RPH3AL

RRAD

RRAGD

RRAS

RRBP1

RRS1

RSAD2

RTN4RL2

RYR1

S100A13

S100A2

S100A3

S100A4

S100A5

S100A8

S100A9

S100B

S100P

SAA1

SAA2

SAA2-SAA4

SAMD4A

SAMD9

SAMD9L

SAMHD1

SAMSN1

SASH3

SBK1

SCARF1

SCCPDH

SCNN1A

SCPEP1

SDC3

SDC4

SDF2L1

SDR16C5

SDS

SDSL

SEC11C

SECTM1

SELENOP

SELPLG

SEMA3E

SEMA4G

SERPINB1

SERPINB9

SERPIND1

SEZ6L2

SFXN3

SGCE

SGK1

SGMS2

SGPP2

SH3RF2

SHC2

SHISA3

SIDT1

SIGLEC1

SIGLEC14

SIK1

SIK1B

SIRPA

SLA

SLAMF8

SLC11A1

SLC14A2

SLC15A1

SLC15A2

SLC15A3

SLC16A14

SLC16A4

SLC16A7

SLC16A9

SLC1A1

SLC1A3

SLC1A4

SLC1A7

SLC20A2

SLC22A18AS

SLC22A23

SLC28A2

SLC28A3

SLC2A5

SLC34A2

SLC35G2

SLC37A2

SLC39A8

SLC40A1

SLC43A3

SLC44A5

SLC45A3

SLC45A4

SLC50A1

SLC5A8

SLC6A20

SLC7A10

SLC7A2

SLC7A7

SLCO2A1

SLCO2B1

SLFN11

SLFN5

SMARCA1

SMARCA4

SMIM22

SMIM3

SMOC1

SMPDL3A

SMURF2

SNX10

SNX21

SOCS1

SOCS3

SOX12

SOX4

SP5

SPATC1L

SPDYC

SPI1

SPINK13

SPINK2

SPINK4

SPN

SPOCK3

SPP1

SPP2

SPRED1

SPRR1A

SPRR1B

SPRR2D

SPRR2F

SPRR3

SPSB2

SPTB

SPTBN2

SPTSSA

SPX

SQOR

SRGN

SRMS

SSC4D

SST

SSX1

ST8SIA4

STAB1

STAC

STARD10

STEAP2

STEAP3

STING1

STK17A

STK32A

STK39

STMN3

STRA6

STS

STUM

STX11

SUCNR1

SULT1C2

SUSD3

SYCE3

SYNPO

SYT12

SYT7

SYT8

SYTL2

TAC3

TACC2

TACSTD2

TBC1D8

TBL1X

TBX15

TBXAS1

TC2N

TCN2

TDRD5

TDRD9

TENM1

TESC

TFAP2C

TFF1

TFPI

TFPI2

TGFA

TGFB1

TGFBI

TGFBR2

TGM2

THBS4

THEM5

THEM6

THEMIS2

THPO

TIMP1

TINCR

TIPARP

TJP3

TKTL1

TLCD1

TLR2

TLR4

TLR8

TM4SF20

TM7SF2

TMED6

TMEM141

TMEM150B

TMEM156

TMEM159

TMEM213

TMEM229A

TMEM233

TMEM256

TMEM45B

TMEM51

TMEM52

TMEM59L

TMEM63C

TMIGD3

TMPRSS13

TMPRSS4

TMSB4X

TMTC1

TNC

TNF

TNFAIP2

TNFAIP8L2

TNFRSF12A

TNFRSF14

TNFRSF18

TNFRSF1B

TNFSF10

TNFSF13

TNFSF13B

TNFSF9

TNIK

TNNI2

TOX

TOX2

TPD52

TPSAB1

TPSB2

TPSD1

TREM1

TREM2

TRIB1

TRIM2

TRIM22

TRIM29

TRIM47

TRIP6

TRPM2

TRPV2

TSC22D3

TSPAN12

TSPAN31

TSPO

TTC39A

TTC39C

TTR

TUBA1A

TUBB2A

TUFT1

TXNDC17

TXNIP

TYMP

TYROBP

UBA7

UBE2L6

UGT2B4

UGT8

ULBP2

UNC13D

UPK1B

UPK3A

UPP1

VANGL2

VAV1

VDR

VIM

VMO1

VNN1

VNN2

VSIG1

VSIG4

VSTM2L

WAS

WDR66

WDR72

WFDC10B

WFDC12

WFDC3

WFDC5

WLS

WNK1

WNT10A

WNT3

WNT4

WNT7B

XAF1

XAGE2

XDH

XK

XKRX

ZC3H12A

ZFP36L1

ZFP36L2

ZFP42

ZNF219

ZNF331

ZNF385A

ZNF486

ZNF626

ZNF710

ZSCAN31

ZYX
